# *Cryptomyrus*: a new genus of Mormyridae (Teleostei, Osteoglossomorpha) with two new species from Gabon, West-Central Africa

**DOI:** 10.3897/zookeys.561.7137

**Published:** 2016-02-08

**Authors:** John P. Sullivan, Sébastien Lavoué, Carl D. Hopkins

**Affiliations:** 1 Cornell University Museum of Vertebrates, 159 Sapsucker Woods Road, Ithaca, New York 14850 USA; 2 Institute of Oceanography, National Taiwan University, Roosevelt Road, Taipei 10617, Taiwan; 3 Department of Neurobiology and Behavior, Cornell University, Ithaca, New York 14853 USA

**Keywords:** Weakly electric fish, Mormyrinae, integrative taxonomy, phylogeny, DNA, electric organ discharge, EOD, African freshwater fishes, rarity

## Abstract

We use mitochondrial and nuclear sequence data to show that three weakly electric mormyrid fish specimens collected at three widely separated localities in Gabon, Africa over a 13-year period represent an unrecognized lineage within the subfamily Mormyrinae and determine its phylogenetic position with respect to other taxa. We describe these three specimens as a new genus containing two new species. *Cryptomyrus*, new genus, is readily distinguished from all other mormyrid genera by a combination of features of squamation, morphometrics, and dental attributes. *Cryptomyrus ogoouensis*, new species, is differentiated from its single congener, *Cryptomyrus ona*, new species, by the possession of an anal-fin origin located well in advance of the dorsal fin, a narrow caudal peduncle and caudal-fin lobes nearly as long as the peduncle. In *Cryptomyrus ona*, the anal-fin origin is located only slightly in advance of the dorsal fin, the caudal peduncle is deep and the caudal-fin lobes considerably shorter than the peduncle. Continued discovery of new taxa within the “Lower Guinea Clade” of Mormyridae highlights the incompleteness of our knowledge of fish diversity in West-Central Africa. We present a revised key to the mormyrid genera of Lower Guinea.

## Abstract (fr)

Nous avons démontré avec des marqueurs mitochondriaux et nucléaires que trois spécimens de poissons mormyridés faiblement électriques, collectés à trois localités au Gabon sur une période de 13 ans, forment une lignée inconnue au sein de la sous famille des Mormyrinae dont nous avons déterminé la position phylogénétique. Nous décrivons un nouveau genre et deux nouvelles espèces pour ces trois spécimens. *Cryptomyrus*, nouveau genre, se distingue des autres genres de mormyridés par une combinaison de caractères de l’écaillure, morphométriques, et dentaires. *Cryptomyrus ogoouensis*, nouvelle espèce, se distingue de sa seule espèce congénérique par sa nageoire anale qui commence nettement en avant de sa nageoire dorsale, son pédoncule caudal gracile et les lobes de sa nageoire caudale pratiquement aussi longs que son pédoncule caudal. Chez *Cryptomyrus ona*, nouvelle espèce, la nageoire anale commence légèrement en avant de l’origine de la nageoire dorsale, le pédoncule caudal est large, et les lobes de la nageoire caudale sont plus courts que le pédoncule caudal. La découverte ininterrompue de nouveaux taxa dans le «clade de basse Guinée» des Mormyridae illustre notre connaissance incomplète de la diversité des poissons de l’ouest de l’Afrique centrale. Nous présentons une clé révisée des genres de Mormyridae de basse Guinée.

## Introduction

Mormyrids are nocturnally active fishes endemic to the continental freshwaters of Africa that produce weak electric impulses from a muscle-derived organ located in the caudal peduncle, anterior to the caudal fin. Using specialized electroreceptors distributed over the skin, mormyrids sense nearby objects and prey organisms as distortions to their self-produced electric field (von der Emde and Schwarz 2002). The electric organ discharge, or EOD, is also used for communication. In many mormyrids the waveform of each short (0.2–12 millisecond) pulse encodes the species identity and sex of the signaler while patterns in the timing of pulses convey motivational states (Baker et al. 2013; Hopkins 1986, 1999; Hopkins and Bass 1981). Due to their frequent species-specificity, recorded EOD waveforms can provide valuable characters for the taxonomy of these fishes (Sullivan et al. 2002; Arnegard and Hopkins 2003; Hopkins et al. 2007). There are currently 223 valid species of Mormyridae placed in 20 genera (Sullivan and Lavoué 2015). The division of Mormyridae into two subfamilies, Mormyrinae (19 genera) and Petrocephalinae (one genus), is supported by both morphological and molecular evidence (Taverne 1972; Sullivan et al. 2000; Lavoué et al. 2003).

Here we describe two new species and a new genus of Mormyrinae, based on only three specimens collected over a period of 13 years at three widely separated localities in Gabon, West Central Africa. Despite significant fish collection effort at a number of sites in Gabon since the late 1990s often specifically targeting mormyrid fishes, we know of no other specimens belonging to this unrecognized mormyrid lineage in museum collections. The fishing effort required to produce these three individuals suggests that these species may be extremely rare in nature or that their precise habitat has yet to be discovered. In either case, we have little confidence that additional material will become available soon and believe description of these taxa should not be further postponed.

**Figure 1.**
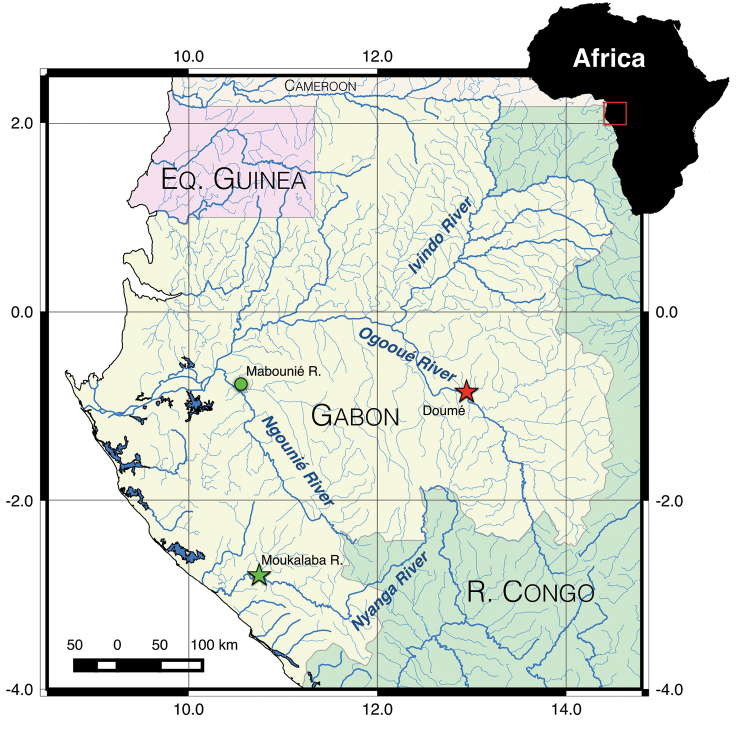
Geographic location of collection sites for the three mormyrid specimens treated in this study. Red star = type locality of *Cryptomyrus ogoouensis* sp. n. at Doumé, Ogooué River; green star = type locality of *Cryptomyrus ona* sp. n. at the Moukalaba River; green circle = locality of second specimen of *Cryptomyrus ona* at the Mabounié River.

**Figure 2.**
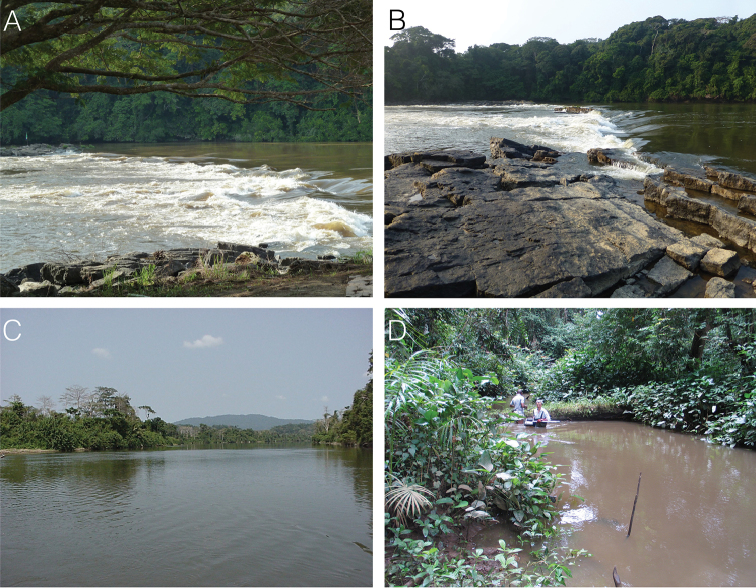
Photographs of the collection localities of the three mormyrid specimens treated in this study. **A** Doumé falls on the Ogooué River, Ogooué-Lolo, Gabon, type locality of *Cryptomyrus ogoouensis* sp. n. during high water in May 2011 **B** same locality in low water, September 2014 **C** Nyanga River at confluence with Moukalaba River near collection site of holotype of *Cryptomyrus ona* sp. n., July 2001 **D** Collection site of *Cryptomyrus ona* specimen CUMV 98647 in Mabounié River, Ngounié Province, February 2012.

In July 2001 the first specimen (MNHN 2003-0425) was collected in a gill net placed in the Moukalaba River close to its confluence with the Nyanga River in southern Gabon (Figs 1, 2). No EOD was recorded, but a tissue sample was taken. The morphological distinctiveness of this fish was noted, but a second trip to the locality was unsuccessful in collecting more specimens. The head, body shape, and nearly equal median fins of the Moukalaba River specimen reminded us of *Hippopotamyrus castor* Pappenheim, 1906 from the Lokoundjé and Sanaga Rivers of Cameroon and to the Nilo-Sudanic species *Hippopotamyrus pictus* (Marcusen, 1864). However, an unpublished phylogenetic analysis of DNA sequences from the mitochondrial 12S, 16S, and cytochrome *b* genes added to a matrix of sequences from other mormyrid taxa including *Hippopotamyrus castor* and *Hippopotamyrus pictus* did not support a close relationship with *Hippopotamyrus*. Description of this fish was deferred in the hope that more specimens would become available for study.

In 2012 a second specimen resembling the Moukalaba River fish was collected in a gill net sample from the Mabounié River, a small, right-bank tributary of the lower Ngounié River (Ogooué basin, Ngounié Province; Figs 1, 2). The specimen was photographed and preserved in ethanol enabling DNA extraction.

Finally, in September 2014 during the course of an ichthyofaunal survey sponsored by The Nature Conservancy in the “Rapids of Mboungou Badouma and Doumé” Ramsar site located along the Ogooué River upstream of Lastoursville (Ogooué-Lolo Province), a single live specimen somewhat resembling the two other fish was collected in an earthworm-baited fish trap at Doumé falls, just beside the village of Doumé (Figs 1, 2). EOD recordings, photographs, and a tissue sample were obtained.

## Materials and methods

As detailed above, the three specimens described here were collected on three separate expeditions to Gabon in 2001, 2012 and 2014. Specimens of other species used in this study are listed below in the comparative material examined section. Institutional abbreviations follow Sabaj Pérez (2014).

### Specimen handling and EOD recording

Specimens were collected, handled and euthanized in accordance with guidelines published by the Use of Fishes in Research Committee (1987, 2004, 2014). EODs of the Doumé specimen (tag no. JPS-1194) were recorded in a small aquarium filled with water from the collection site, using chloridized-silver wire electrodes connected to an Echo 2 USB analog to digital converter (Echo Audio, Inc.) sampling at 192 kHz/16 bits. We visualized and saved signals using SignalScope virtual oscilloscope software (Faber Acoustical, LLC). We recorded head positivity of the fish in the upward direction and noted water temperature at time of recording. After recording, the fish was euthanized with an overdose of the anesthetic MS222 (tricaine methanesulfonate), tagged, and fixed in 10% formalin. The specimen was subsequently transferred to 70% ethanol and is deposited in the Cornell University Museum of Vertebrates as CUMV 98155.

### DNA sequencing and phylogenetic analysis

In order to investigate the phylogenetic relationships of the three novel mormyrid specimens, we sequenced from each the complete cytochrome *b* (cyt-*b*) gene and portions of the 12S and 16S genes from the mitochondrial genome, as well as partial *rag*2 and the complete S7 introns 1 & 2 and the short intervening exon from the nuclear genome. We added these sequences to an existing 4209 bp alignment of these markers from 38 species belonging to 17 mormyrin genera published in Lavoué et al. (2003), the most complete molecular phylogenetic study of the Mormyrinae to date. In addition to these markers used for phylogenetic analysis, we sequenced a 636 bp fragment of the cytochrome oxidase I (COI) gene, the “barcode” marker from the three specimens for comparison to sequences found in the Barcode of Life Database (Ratnasingham and Hebert 2007).

Because the possible relationship of these new taxa to the genus *Hippopotamyrus* Pappenheim, 1906 was of special interest, we also sequenced all of these markers from a specimen of *Hippopotamyrus castor* from Cameroon, the type species of this genus, a species not included in Lavoué et al. (2003). In addition, we added to the expanded matrix cyt-*b* sequences of a specimen of *Hippopotamyrus pictus* from the White Nile of Ethiopia (complete data for a *Hippopotamyrus pictus* from the Niger basin of West Africa was already included in the dataset) as well as cyt-*b* sequences retrieved from GenBank for *Hippopotamyrus ansorgii* (Boulenger, 1905) and *Hippopotamyrus szaboi* Kramer, van der Bank & Wink, 2004, coding missing data as “?” in the matrix.

DNA was extracted from fin clips or epaxial muscle tissue preserved in 95% ethanol using a QIAGEN (QIAGEN, Inc., Valencia, CA) DNeasy kit. Primer sequences for cyt-*b*, 12S, 16S, *rag*2, S7 and COI are listed in Table 1. For each marker we carried out PCR in 10 to 30μl reactions with components at the following concentrations: 1x Sigma PCR buffer (Sigma–Aldrich, St. Louis, MO), 0.02 U/μl Sigma JumpStart *Taq*, 2 mM MgCl_2_ (3 mM MgCl_2_ for *rag*2), 0.4 μM of forward and reverse primer, 200 μM of each dNTP and approximately 200 pg/μl template DNA. We used an initial denaturation step of 1 min at 94 °C followed by 35 cycles of 94 °C for 30 s, annealing at 54 °C (59 °C for *rag*2) for 30 s, and extension at 72 °C for 1.5 min, followed by a final 72 °C extension step for 10 min. We evaluated amplification success on ethidium bromide-stained agarose gels, purified PCR products using Exonuclease I and Shrimp Alkaline Phosphatase and dye-deoxy termination cycle sequencing using ABI Big Dye chemistry followed by Sephadex column purification and data collection on an Applied Biosystems Automated 3730xl analyzer (PE Applied Biosystems, Foster City, CA).

**Table 1.** Forward and reverse primers used to amplify six genetic markers used in this study shown in 5’ to 3’ orientation.

cytbF-L14724	GAC TTG AAA AAC CAC CGT TG
cytbR-H15915	CTC CGA TCT CCG GAT TAC AAG AC
COIF-ZPeng	TCT CAA CCA ACC ATA AAG ACA TTG G
COIR-ZPeng	TAT ACT TCT GGG TGC CCA AAG AAT CA
12S-L1067	AAA CTG GGA TTA GAT ACC CCA CTA T
12S-H1478	GAG GGT GAC GGG CGG GCG GTG TGT
16S-L2510	CGC CTG TTT ATC AAA AAC AT
16S-H3080	CCG GTC TGA ACT CAG ATC ACG T
rag2F2	ArA CGC TCm TGT CCm ACT GG
rag2R6	TGr TCC ArG CAG AAG TAC TTG
S7RPEX1F	TGG CCT CTT CCT TGG CCG TC
S7RPEX3Ralt	ACC TTT GCT GCA GTG ATG TT

Sequences were edited and combined into contigs for each fragment with Sequencher 4.2 (GeneCodes Corporation, Ann Arbor, MI). Requiring no gaps, alignment of the new coding gene sequences (cyt-*b* and *rag*2) to the Lavoué et al. (2003) dataset was trivial; for 12S, 16S and S7 we were able to align these sequences by eye to the previously optimized alignment without need for insertion of additional gaps.

We performed a maximum likelihood phylogenetic analysis of the matrix from Lavoué et al. (2003) with the added taxa in RAxML v.8, implemented on XSEDE (Stamatakis 2014) via the CIPRES Science Gateway web server (Miller et al. 2010) using separate GTRGAMMA evolutionary models for each gene partition and performed a non-parametric bootstrap analysis to estimate support for nodes. Bootstrapping was auto-terminated using the autoMRE criterion. All other settings were left at their default values. The tree was rooted using *Myomyrus macrops* Boulenger, 1914 as outgroup (Lavoué et al. 2003).

### Morphometrics and meristics

**Figure 3.**
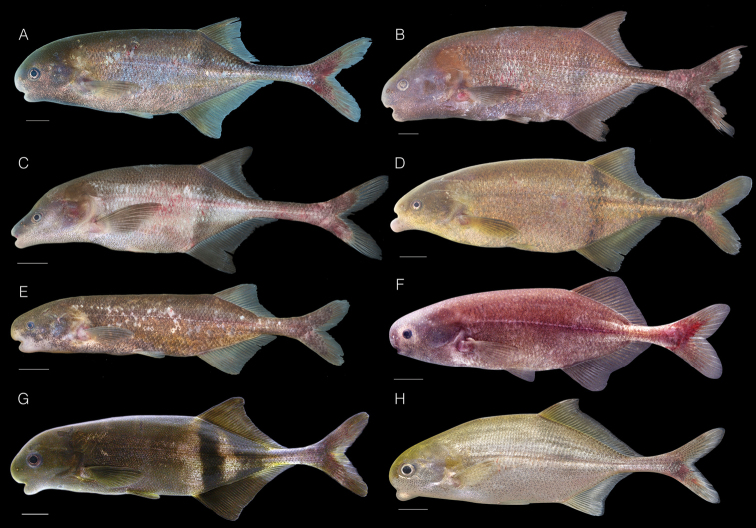
Some mormyrid species used for morphological comparison in this study. **A** *Ivindomyrus marchei* female, 135 mm SL, CUMV 98172, tag no. JPS-1233, Sébé River, Ogooué-Lolo, Gabon **B** *Ivindomyrus opdenboschi* female, 160 mm SL, CUMV 96829, tag no. JPS-1057, Ivindo River at Loa Loa, Ogooué-Ivindo, Gabon **C** *Boulengeromyrus knoepffleri* juvenile 107 mm SL, CUMV 96838, tag no. JPS-1055, Ivindo River at Loa Loa, Ogooué-Ivindo, Gabon **D** *Marcusenius moorii* female, 147 mm SL, CUMV 96836, tag no. JPS-1110, Ogooué River at Franceville, Haut-Ogooué, Gabon **E** *Paramormyrops* sp. (undescribed species) female, 100 mm SL, CUMV 98119, tag no. JPS-1148, Moumba Creek, Ogooué-Lolo, Gabon **F** *Stomatorhinus walkeri* female 95 mm SL, CUMV 80227, tag no. 2814, Diengui Creek, Moyen-Ogooué, Gabon **G** *Hippopotamyrus castor* female, 122 mm SL, CUMV 89955, tag no. 6018, Sanaga River at Nachtigal Falls, Centre, Cameroon **H** *Cyphomyrus psittacus* female, 109 mm SL, CUMV 96767, tag no. JPS-0438, Congo River at Yangambi, Orientale, D.R. Congo.

Because the three specimens under study do not obviously belong to any one of the 19 described mormyrin genera, choosing mormyrin species for comparison was not straightforward. We collected counts and measurements from seven sympatric taxa with which the new taxa could conceivably be confused in the field and two extralimital taxa with (in our estimation) the most overall resemblance to the three specimens, both of which are type species of genera not known to occur in Gabon. The sympatric taxa used for comparison are *Ivindomyrus marchei* (Sauvage, 1879), *Ivindomyrus opdenboschi* Taverne & Géry, 1975, *Marcusenius moorii* (Günther, 1867), *Stomatorhinus walkeri* (Günther, 1867), *Paramormyrops kingsleyae* (Günther, 1896) and two undescribed species of *Paramormyrops* Taverne, Thys van den Audenaerde & Heymer, 1977. The extralimital taxa used for comparison are *Hippopotamyrus castor* (from coastal drainages of Cameroon) and *Cyphomyrus psittacus* (Boulenger, 1897) from the Congo basin. Most of these species are illustrated in Figure 3.

We took 29 point-to-point measurements with a digital caliper on each of the three specimens under study for comparison to those taken from 115 individuals of nine species listed above. Twenty-seven of these measurements are defined in Boden et al. (1997): total length (TL), standard length (SL), body depth (BD), caudal peduncle depth (CPD), middle caudal peduncle depth (MCPD), caudal peduncle length (CPL), head length (HL), head depth (HD), head width (HW), snout length (SNL), interorbital width (IOW), eye diameter (ED), postorbital length (POL), internarine distance (DNN), posterior nares-eye distance (DNE), predorsal distance (PDD), preanal distance (PAD), prepelvic distance (PPLD), prepectoral distance (PPCD), dorsal-fin length (DFL), dorsal-fin height (DFH), anal-fin length (AFL), anal-fin height (AFH), pelvic-fin length (PLFL), pectoral-fin length (PCFL), pelvic-to-anal-fin distance (DPLAF), and pectoral-to-anal-fin distance (DPCAF). To these we added two additional measures: head length to the end of the opercle bone (HLBO), and mouth width measured at the inner corner of the front of the mouth (MW).

To facilitate dorsal- and anal-fin ray comparisons among individuals, we report the number of unbranched rays (3) + number of branched rays. (The first two rays are small, the first often only visible in radiographs.)

Specimens were radiographed using a Faxitron Model LX-60 cabinet x-ray system and Kodak Industrex MX125 film. Film images were scanned on a flatbed scanner.

We follow Taverne (1968) in reporting features of the axial skeleton although we exclude the demi-centrum fused to hypurals 3+4 in our vertebral counts. Our nomenclature for hypural bones follows Teugels and Hopkins (1998) and that for intermuscular bones follows Patterson and Johnson (1995).

Sex of the specimens was determined by presence/absence of an “anal-fin notch”: a dorsally directed indentation along the anterior base of the anal fin present in all mature male mormyrids (Pezzanite and Moller 1998).

## Results

### EOD recording of the Doumé specimen

**Figure 4.**
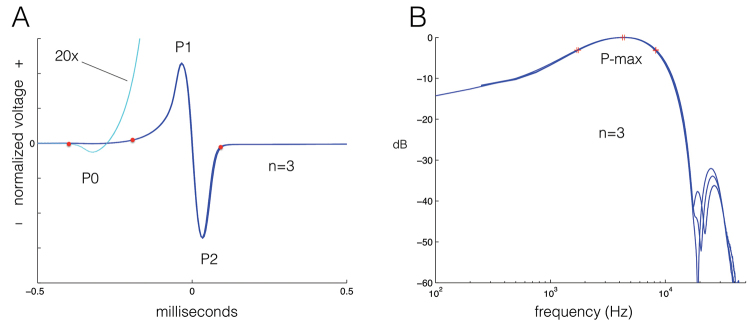
**A** Three superimposed electric organ discharge (EOD) waveform recordings of *Cryptomyrus ogoouensis* holotype CUMV 98155 (Macaulay Library #197475) recorded at 23.2 °C, head positivity upwards, X-axis = 1 millisecond, Y-axis tick marks indicate 20% of EOD peak-to-peak height; P0, P1, P2 mark the positive and negative excursions in the waveform, P0 highlighted by 20× amplification; red dots indicate onset of P0, onset of P1 and offset of P2, respectively **B** Power spectrum of EOD waveforms in A; red “+” symbols mark peak frequency and frequencies -3 dB below peak at 4300 Hz.

The EOD waveform of the specimen from Doumé is triphasic and very brief (Fig. 4A). Among mormyrids of Lower Guinea, this EOD is most similar to that recorded from *Hippopotamyrus castor* from Cameroon (Hopkins et al. 2007), but dissimilar to that of every other species recorded so far in Gabon. Details of the EOD are provided in the species description below, and the recording is available in the archive of the Macaulay Library at the Cornell Lab of Ornithology under accession number ML197475.

### DNA sequences

GenBank numbers for the new sequences generated for this study and their appropriate GenSeq codes (Chakrabarty et al. 2013) are given in Table 2. The sequence data (Table 3) confirm that the three specimens are very close relatives while genetically distant from other mormyrins. The cyt-*b* sequences of the Doumé and Moukalaba specimens differ at 15 sites across the 1140 base pairs, or a p-distance of 1.32%. Between the Doumé specimen and the Mabounié specimen this difference is 17 sites or 1.49%. Between the Moukalaba and the Mabounié specimens the difference is only six sites, or 0.53%. COI genetic distance is smaller between the three specimens: a p-distance of only 0.6% between the Doumé and Moukalaba specimens, 0.8% between the Doumé and the Mabounié specimen, and 0.2% between the Moukalaba and Mabounié specimens. Genetic distances for the 12S and 16S markers are similar to COI, and nuclear *rag*2 and S7 sequences were identical among all three (Table 3) with the exception of base 564 in the S7 sequence which is heterozygous for C and T in the Doumé specimen (coded as “Y” in the sequence), while homozygous for T in the other two specimens. For all markers, sequences from the two species of *Ivindomyrus* Taverne & Géry, 1975 and from *Boulengeromyrus knoepffleri* Taverne & Géry, 1968 are closest to those of the new taxa, with cyt-*b* p-distances between 6.1 and 6.5% (Table 3).

**Table 2.** GenBank numbers and specimen information for DNA sequences generated in this study. All but COI were added to alignment of Lavoué et al. (2003) to investigate phylogenetic relationships of new taxa. GenSeq nomenclature follows Chakrabarty et al. (2013).

Specimen	Catalog no.	COI	cyt-*b*	12S	16S	rag2	S7	GenSeq Status
*Cryptomyrus ogoouensis* holotype	CUMV 98155	KT875221	KT875226	KT875213	KT875217	KT875230	KT875233	genseq-1 COI, cyt-*b*, 12S, 16S, *rag*2, S7
*Cryptomyrus ona* holotype	MNHN 2003-0425	KT875222	KT875227	KT875214	KT875218	KT875231	KT875235	genseq-1 COI, cyt-*b*, 12S, 16S, *rag*2, S7
*Cryptomyrus ona* Mabounié specimen	CUMV 98647	KT875223	KT875228	KT875215	KT875219	KT875232	KT875234	genseq-3 COI, cyt-*b*, 12S, 16S, *rag*2, S7
*Hippopotamyrus castor*	CUMV 89959-6072	KT875220	KT875224	KT875212	KT875216	KT875229	KT875236	genseq-3 COI, cyt-*b*, 12S, 16S, *rag*2, S7
*Hippopotamyrus pictus* (White Nile)	CUMV 94598-1159		KT875225					genseq-4 cyt-*b*

**Table 3.** Genetic distances (uncorrected p-distances) between three *Cryptomyrus* specimens and nearest relatives *Boulengeromyrus knoepffleri* and *Ivindomyrus marchei* for mitochondrial and nuclear markers sequenced, shown in order as COI/cyt-*b*/combined 12S-16S/rag2/S7. *Boulengeromyrus knoepffleri* CUMV 92903 and *Ivindomyrus marchei* CUMV 92346 used for COI comparison.

1	*Boulengeromyrus knoepffleri*	1	2	3	4	5
2	*Ivindomyrus marchei*	0.033/0.051/0.014/0.004/0.011	•			
3	*Cryptomyrus ogoouensis* holotype	0.085/0.065/0.028/0.022/0.021	0.083/0.064/0.025/0.017/0.015	•		
4	*Cryptomyrus ona* holotype	0.085/0.063/0.028/0.021/0.021	0.080/0.064/0.028/0.017/0.015	0.006/0.013/0.006/0.0/0.0	•	
5	*Cryptomyrus ona* Mabounié specimen	0.083/0.061/0.027/0.021/0.021	0.079/0.064/0.026/0.017/0.015	0.008/0.015/0.005/0.0/0.0	0.002/0.005/0.001/0.0/0.0	•

We note that genetic differentiation observed between the Doumé specimen and the Moukalaba and Mabounié specimens, while small, is within the range seen between some closely related, but distinct mormyrid species. For example cyt-*b* sequences from the morphologically divergent *Paramormyrops gabonensis* Taverne, Thys van den Audenaerde & Heymer, 1977 and *Paramormyrops hopkinsi* Taverne & Thys van den Audenaerde, 1985 – two species for which there is no evidence of mitochondrial introgression – similarly differ by 1.3%. However, in other cases, populations from different river basins regarded as conspecific can differ by this much. For example, *Ivindomyrus marchei* from the Ogooué and Ivindo Rivers differ from those in the Nyanga River by just slightly less than 1.3% (Lavoué et al. 2008) as do Congo and Ogooué basin populations of *Marcusenius moorii* (unpublished data). The degree of genetic differentiation between the Moukalaba River and Mabounié River specimen (0.53%), on the other hand, is squarely within the normal range of intraspecific variation for cyt-*b*, with the exception of cases where introgressive hybridization is suspected.

Among the more than 300 mormyrid COI sequences in the Barcode of Life Database (BOLD; Ratnasingham and Hebert 2007), the closest match to those from the new taxa is from an *Ivindomyrus marchei*, with 92% sequence identity. To further explore the relationship of mitochondrial sequence differentiation within and between mormyrid species, we downloaded all of the mormyrid COI sequences archived in BOLD and calculated intraspecific variation in COI for species represented by more than one sequence. Intraspecific distances in the COI dataset are well above the 0.6–0.8% observed between the Doumé specimen and the other two individuals for several nominal species with wide distributions spanning multiple major watersheds, e.g. *Brevimyrus niger* (Günther, 1866), *Gnathonemus petersii* (Günther, 1862), and *Mormyrops anguilloides* (Linnaeus, 1758). However, there are also examples of species pairs with COI sequence differentiation below 1% p-distance within genera *Marcusenius* Gill, 1862, *Campylomormyrus* Bleeker, 1874, *Cyphomyrus* Myers, 1960, and *Petrocephalus* Marcusen, 1854. While some of these findings could be attributed to identification errors and/or mitochondrial introgression between sympatric species, morphologically distinct species with very low mitochondrial genetic divergence are documented within the riverine species flocks of *Campylomormyrus* (Feulner et al. 2007) and *Paramormyrops* (Sullivan et al. 2002, 2004).

Turning to the nuclear markers, the near identity of the nuclear S7 intron sequences among the Mabounié, Doumé and Moukalaba specimens might be thought surprising if these three specimens represent more than one species (less surprising for the identity of the *rag*2 sequences, as this marker evolves more slowly). However, distinct mormyrid species having identical or near-identical nuclear S7 intron sequences is not unprecedented, as similarly identical S7 intron sequences were found among three species of *Campylomormyrus* by Feulner et al. (2007).

### Phylogenetics

The aligned dataset and tree described here have been archived on TreeBASE and can be accessed at http://purl.org/phylo/treebase/phylows/study/TB2:S18468?format=html.

**Figure 5.**
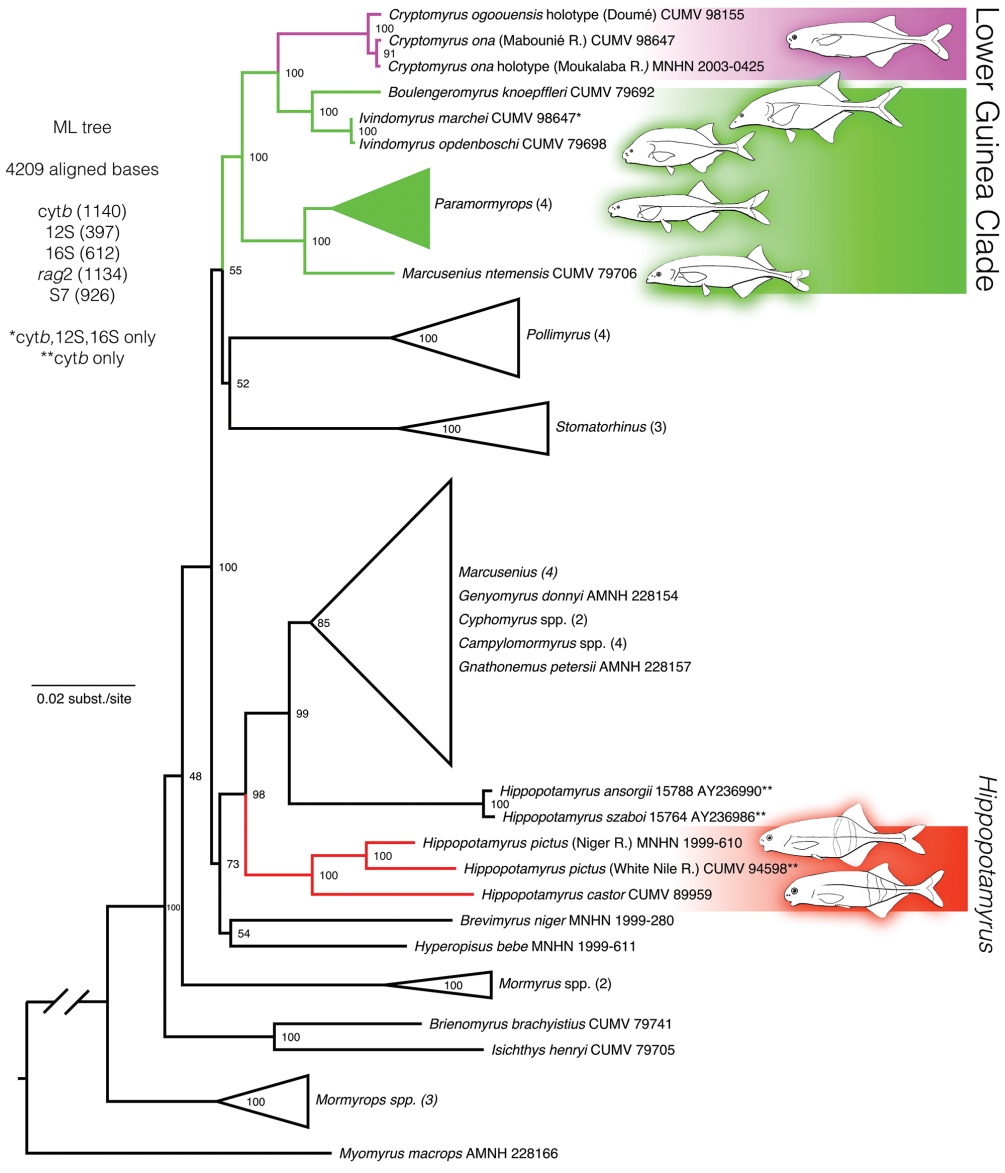
Maximum likelihood phylogenetic tree calculated in RAxML for 46 mormyrin specimens based on DNA sequences from mitochondrial cytochrome *b*, 12S, 16S and nuclear *rag*2 and S7 intron, rooted with *Myomyrus macrops*. Bootstrap support values at nodes. Some clades collapsed for simplicity. Position of new taxa (magenta) within the “Lower Guinea Clade” (green) and distance from *Hippopotamyrus castor*, type species of genus *Hippopotamyrus* (red), highlighted.

In the maximum likelihood tree produced in RAxML (Fig. 5) the three specimens form a monophyletic group, with the Moukalaba River and Mabounié River specimens paired together, sister to the Doumé specimen, as one would predict from the genetic distances. Together, the three specimens appear as the sister lineage to the clade formed by *Boulengeromyrus knoepffleri* plus the two *Ivindomyrus* species. All of these together are in turn sister to the clade formed by species of *Paramormyrops* plus *Marcusenius ntemensis* (Pellegrin, 1927). Bootstrap support at all of these nodes is 100% with the exception of a 91% value at the node joining the Moukalaba River and Mabounié River specimens. This larger clade that includes taxa either endemic to or most diverse in Lower Guinea is sister to a clade formed by species of genus *Stomatorhinus* Boulenger, 1898 plus species of genus *Pollimyrus* Taverne, 1971, with lower bootstrap support. *Hippopotamyrus castor* from Cameroon, the type species of *Hippopotamyrus*, clusters far separately from the new taxa from Gabon, sister to two *Hippopotamyrus pictus* individuals, one from the White Nile River and one from the Niger River. These are sister to a large clade containing species of *Marcusenius*, *Campylomormyrus*, *Gnathonemus* Gill, 1863, *Cyphomyrus*, *Genyomyrus* Boulenger, 1898 and a separate pair of species from southern Africa classified as *Hippopotamyrus*: *Hippopotamyrus ansorgii* and *Hippopotamyrus szaboi*. While incidental to the description of the new taxa treated here, this result implies the “*Hippopotamyrus ansorgii* complex” of southern Africa treated by Kramer et al. (2004) and Kramer and Swartz (2010) does not belong in *Hippopotamyrus* Pappenheim and requires classification in a different genus.

To explore the relative contribution of mitochondrial and nuclear data to the phylogenetic result, we ran two additional analyses, one with only mitochondrial data included for the three specimens and the other with only nuclear data included for them. In each case, excluded bases were recoded as missing data, while the full dataset was used for all other specimens. In the nuclear data-only analysis, we used a single OTU for the three specimens since *rag*2 and S7 sequences were identical among them. In both experimental analyses, the lineage of the three new taxa was resolved as sister to *Boulengeromyrus* plus *Ivindomyrus* with a 100% bootstrap proportion, indicating strong signal contributing to this result from both the nuclear and mitochondrial data partitions.

### Morphometrics and meristics

Consistent with the genetic distances reported above, measures and counts of the Moukalaba and Mabounié specimens are more similar to each other than they are to those of the Doumé specimen. In particular, dorsal- and anal-fin bases are short in the former two, with low fin-ray counts, 20/25 and 21/24 total rays respectively, compared to 24/30 in the Doumé specimen, in which the anal-fin origin is situated much further in advance of the dorsal-fin origin. Predorsal distance is nearly equal to preanal distance in the Moukalaba and Mabounié specimens, while it is markedly shorter in the Doumé specimen due to its longer anal fin. Additionally, the caudal peduncle is much deeper and the caudal-fin lobes are shorter in the Moukalaba and Mabounié specimens than in the Doumé specimen. We have never observed a range of four dorsal-fin rays, six anal-fin rays, and differences of 25 percent in anal-fin length relative to standard length among individuals of a single mormyrid species.

**Figure 6.**
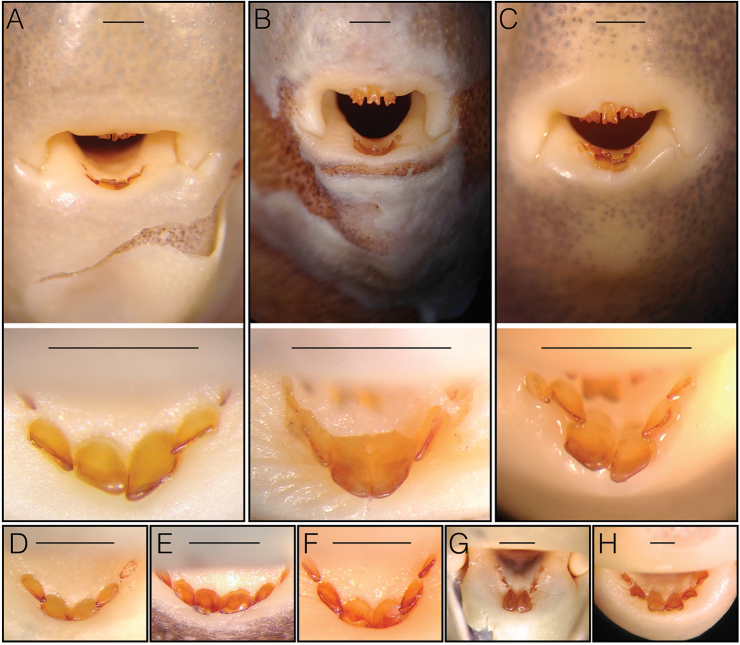
Mouth and dentary teeth in **A** *Cryptomyrus ogoouensis* holotype CUMV 98155 **B** *Cryptomyrus ona* holotype MNHN 2003-0425 **C** *Cryptomyrus ona* non-type CUMV 9864; dentary teeth in **D** *Boulengeromyrus knoepffleri* CUMV 81643 tag no. 2254 **E** *Ivindomyrus marchei* CUMV 96827 tag no. JPS-1043 **F** *Ivindomyrus opdenboschi* CUMV 89324 tag no. 5654 **G** *Hippopotamyrus castor* CUMV 89955 tag no. 6033 **H** *Paramormyrops* sp. “SN4” CUMV 81322. Scale bars = 1 millimeter.

While our initial hypothesis that the new taxa are close relatives of *Hippopotamyrus castor* from Cameroon was ruled out by the molecular result, substantial morphological differences are also obvious between the new taxa and *Hippopotamyrus castor*. The new taxa have fewer scales (44–45 lateral line scales/12 around the caudal peduncle vs. 72–81/16 in *Hippopotamyrus castor*), fewer total vertebrae (40–43 vs. 47), fewer dorsal-fin rays (20–24 vs. 31–33), and a longer anal-fin base (dorsal 78–88% of anal-fin base vs. nearly equal), among other significant differences. Also, while *Hippopotamyrus castor* has elongate paired incisor teeth in the lower jaw (from which its specific epithet derives), their morphology is quite different from the lower teeth of *Cryptomyrus*. In the three specimens under study, the central incisors are flattened and spatulate. The neighboring pair of teeth are smaller, but similarly spatulate and close or appressed to the inner pair which assume a sort of trowel shape, pointing outward (Fig. 6). In *Hippopotamyrus castor*, the central incisors are cylindrical and club-like and the lateral teeth are greatly reduced (Fig. 6).

With its moderately swollen chin and small mouth, the head of *Cyphomyrus psittacus* appears somewhat similar to those of the new taxa and like them *Cyphomyrus psittacus* has a diffuse band of dark pigment between the origin of the dorsal and anal fins, but the new taxa differ from *Cyphomyrus psittacus* in being less deep bodied (BD 21–24% SL vs. 30–34%), having a shallower head (HD close to 79% HL vs. 63–75%), a smaller eye (ED 19–20% HL vs. 24–26%), a dorsal fin somewhat shorter than the anal fin (vs. far longer than the anal fin) and fewer midlateral scales (44–45 vs. 54–56). Unlike the spatulate dentary teeth of the new taxa, dentary teeth in species of *Cyphomyrus* examined are very small, feebly notched, and embedded in the gums.

The three specimens also differ morphologically from individuals of the two species of *Ivindomyrus* to which the phylogenetic study shows they are closely related. Compared to *Ivindomyrus*, all three specimens have shorter, deeper caudal peduncles (CPD 31–35% CPL vs. 21–28%), greater interorbital width compared to head length (IOW 33–35% HL vs. 21–30%), a higher dorsal-fin to anal-fin length ratio (DFL 78–83% AFL vs. 65–76%), and considerably fewer midlateral scales (44–45 vs. 53–58). Like the dentary teeth in the new taxa, those in *Ivindomyrus* are spatulate, however the central pair of incisors are not noticeably elongate along their inner margins as they are in the new taxa (Fig. 6).

Compared to species of *Paramormyrops* the new taxa are generally deeper bodied (BD 21–25% SL vs. 15–21%) with deeper heads (HD close to 79% HL vs. 63–75%), larger eyes (ED 19% HL vs. 9–17%) and shorter postorbital head length (POL 59–60% HL vs. 61–70%) in addition to having far fewer midlateral scales (44–45 vs. 56–71). Dentary teeth in all *Paramormyrops* are bicuspid, not spatulate (Fig. 6).

Compared to *Marcusenius moorii*, a species with a similarly low midlateral line scale count, the new taxa most notably lack a chin swelling that protrudes forward beyond the tip of the snout, have a much more subdued and diffuse area of pigment between the dorsal-fin and anal-fin origins, a larger eye (ED 19–20% HL vs. 15–18%) and unicuspid (vs. bicuspid or notched) dentary teeth.

Two of the three new taxa are of larger size than the largest *Stomatorhinus walkeri* examined and all three have a smaller head relative to standard length (HL 22–23% SL vs. 28–31%) a larger eye (ED 19% HL vs. 9–13%), a modest chin swelling (vs. none), and the posterior nares are remote from the rictus of the mouth (vs. in close proximity).

### Generic and specific classification

Based on the molecular phylogenetic analysis, we conclude that these three morphologically distinctive specimens represent an undescribed lineage of Mormyrinae sister to the clade formed by *Boulengeromyrus* plus *Ivindomyrus*. This phylogenetic finding in combination with the morphological differences that exist between these new taxa and *Boulengeromyrus* and *Ivindomyrus* precludes placing them in either genus. Creation of an enlarged *Boulengeromyrus* Taverne & Géry via synonymy in which to subsume all of these taxa is ruled out by the significant morphological differences between them and lack of known morphological synapomorphies. Hence a new genus is required.

We recognize two species: one for the Doumé specimen and the other comprising the Moukalaba and Mabounié specimens, reflecting the greater morphological and genetic similarity of these latter two and their notable morphological differences from the Doumé specimen, discussed above. We choose the Moukalaba River specimen as type for the second species and treat the Mabounié River specimen as a non-type. We describe these new taxa below.

## Taxonomy

### Cryptomyrus gen. n.

Taxon classificationAnimaliaOsteoglossiformesMormyridae

http://zoobank.org/E7F2B850-5FEB-4859-AD41-0DE6A079398C

#### Type species.

*Cryptomyrus ogoouensis* sp. n.

#### Included species.

*Cryptomyrus ogoouensis* sp. n., *Cryptomyrus ona* sp. n.

#### Diagnosis.

*Cryptomyrus* gen. n. is distinguished from all other mormyrid genera by combination of the following features. Scales large: 44 or 45 along the midlateral line, with about 42 pierced lateral line scales; mouth subinferior; broad but nonprotrusive chin swelling that does not extend beyond snout; snout expansive and rounded in lateral profile with slight inflection point visible above anterior margin of eye in lateral view; snout somewhat v-shaped in dorsal view; eye large, 19–20% HL; middle four teeth on dentary squarish, broad and spatulate, oriented nearly horizontally, central two lower teeth longest along inner edges and in contact with each other, jutting in advance of neighboring pair and forming a trowel-like shape (Fig. 6); ventral profile of head with marked concavity between gular region and chin, body depth increasing rapidly from there to pelvic-fin origin, body depth at pelvic-fin origin 21–24% SL; interorbital width 32–35% HL; dorsal-fin length 78–88% of anal-fin length; caudal-peduncle depth at end of anal fin greater than 30% of caudal-peduncle length; faint, wide and diffuse band of pigment between anterior portion of dorsal fin and anal-fin bases, darkest from midlateral region dorsally.

#### Comparisons.

Very few Mormyrinae have so few midlateral scales. Only some of the large-scaled *Marcusenius* such as *Marcusenius moorii* (Boden et al. 1997) and smaller species of *Stomatorhinus* and *Pollimyrus* are in the same range. *Cryptomyrus* has a more pronounced chin swelling than *Ivindomyrus* and *Paramormyrops*, although it does not protrude forward beyond the snout as in *Marcusenius*. *Cryptomyrus* has a noticeably more fusiform body shape and larger eye (19–20% HL) than *Paramormyrops* (eye 11–16% HL), the most speciose mormyrin genus in Gabon and Lower Guinea, which also lacks the band of pigment between the dorsal and anal fins.

#### Etymology.

Gender masculine; from the Greek *kryptos* meaning secret or hidden referring to the rarity of these fishes in collections and the Greek *myros*, a kind of fish, a suffix used in the names of many other mormyrid genera.

### Cryptomyrus ogoouensis sp. n.

Taxon classificationAnimaliaOsteoglossiformesMormyridae

http://zoobank.org/61AA9C32-4368-47D8-98DD-DF7FA8A279CC

Fig. 7 ; Table 4

#### Holotype.

CUMV 98155, tag no. JPS-1194, 132 mm TL, 111.6 mm SL, female; Ogooué–Lolo Province, Gabon: Ogooué River at Doumé falls, off rocks on left bank near village of Doumé, approx. 1.5 meters depth, 0°50.4822'S, 12°57.9288'E, earthworm-baited fish trap, J.P. Sullivan, B. Sidlauskas, J.H. Mve Beh, J. Cutler, & A. Dolé, 17 September 2014.

#### Diagnosis.

*Cryptomyrus ogoouensis* sp. n. is readily differentiated from its sole congener, *Cryptomyrus ona* sp. n., in the possession of an anal-fin origin located well in advance of the dorsal fin (first dorsal ray above anal-fin ray 7 vs. first dorsal ray above anal-fin ray 3), a narrow caudal peduncle (depth 5.1% SL vs. 6.0–6.8% SL), and lobes of caudal fin nearly as long as caudal peduncle (vs. markedly shorter).

#### Description.

Morphometric and meristic data for holotype (female, 111.6 mm SL) presented in Table 4. Maximum size of this species unknown. Body fusiform with dorsal and ventral profiles gently convex, greatest body depth between terminus of pelvic fin and urogenital pore. Body compressed, widest at head. Dorsal head profile very nearly straight from back of head to above eye, a slight indentation above anterior margin of eye and abruptly angled at tip of snout. In lateral view, upper lip to tip of snout nearly a straight line, forming a 90° angle with top of head. Ventral profile of head with marked concavity between gular region and chin, body depth increasing rapidly from there to below mid level of pectoral fin. Snout blunt, deep and broad, rounded from above. Tip of snout on level with center of eye, projecting beyond upper lip. Nostrils well separated from each other and from eye; posterior naris at level of bottom of orbit, anterior naris just below line through center of eye; straight line drawn through nostrils passes through tip of snout and just below pectoral-fin origin. Mouth subinferior, small, rictus beneath posterior naris in advance of eye. Chin swelling present: expansive, but not protruding beyond snout. Premaxillary teeth 5, notched, dentary teeth 6, broad, squarish, spatulate, center four oriented horizontally. Center two projecting beyond neighboring pair, longest along inner edges (Fig. 6). Eye large, laterally positioned on head. Predorsal distance greater than preanal distance; anal-fin origin well in advance of dorsal fin: first dorsal ray above anal-fin ray 7 (branched ray 4); dorsal-fin base shorter than anal-fin base; dorsal-fin rays 3+21, anal-fin rays 3+28; 3 anal-fin rays beyond last dorsal-fin ray. Dorsal fin with strongly falcate posterior margin. Longest dorsal-fin ray is second branched ray; next 10 rays successively shorter; final 8 rays equal in length. Anal fin with gently falcate posterior margin. Longest anal-fin ray is third branched ray; next 13 rays successively shorter, final 12 rays equal in length. Pectoral fin long and pointed distally, with 10 rays, extending to vertical through middle of pelvic fin. Pelvic fin with 6 rays, closer to pectoral-fin origin than to anal-fin origin. Caudal peduncle narrow, depth at middle equal to depth at origin. Caudal fin deeply forked with 20 rays in each lobe, lobes long with rounded ends, scaled at bases; distance from caudal flexion to caudal tips roughly equal to CPL. Body covered by thin, cycloid scales, head naked. Body scales large along sides, smaller dorsally: 43 pierced lateral line scales + 2 unpierced along midlateral line, 19 scales in transverse series between origins of dorsal and anal fins, 9 from pelvic fin to midlateral line, 12 around caudal peduncle. Total vertebrae 43; epineurals associated with vertebrae 1–9, vertebral centra 2–13 with pleural ribs directly attached, vertebrae 14–17 with pleural ribs displaced beneath haemal arches; caudal vertebrae 18–43. Hypurals 1 & 2 unfused.

**Figure 7.**
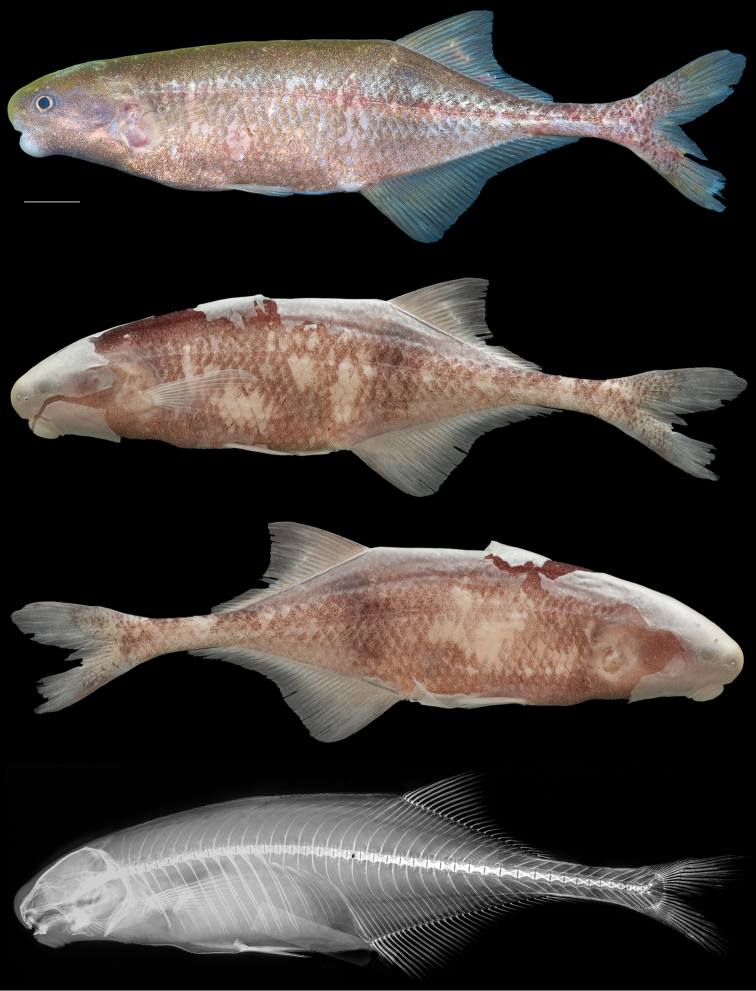
Holotype of *Cryptomyrus ogoouensis* CUMV 98155, female, 112 mm SL, Ogooué River at Doumé falls, Ogooué-Lolo Province, Gabon. Left and right views of preserved whole specimen and radiograph shown below photo of specimen immediately post-mortem. Scale bar = 1 centimeter.

**Table 4.** Descriptive morphometrics and meristics. Data shown for holotype of *Cryptomyrus ogoouensis* sp. n. (CUMV 98155) and the holotype (MNHN 2003-0425) and second specimen (CUMV 98647) of *Cryptomyrus ona* sp. n. expressed in millimeters (mm) and as a percent of standard measures.

Character	*Cryptomyrus ogoouensis* sp. n. Holotype CUMV 98155 Ogooué River at Doumé	*Cryptomyrus ona* sp. n. Holotype MNHN 2003-0425 Moukalaba River	*Cryptomyrus ona* sp. n. Non-type CUMV 98647 Mabounié River
Standard length (SL), mm	111.6	107.8	98.1
Head length (HL), mm	24.3	24.3	22.6
**Percent of SL**			
Body depth at pelvic fin (BD)	23.4	24.2	20.8
Head length (membrane) (HL)	21.8	22.6	23.0
Head length (opercle bone) (HLBO)	18.1	20.3	19.6
Predorsal distance (PDD)	62.7	62.4	63.1
Preanal distance (PAD)	55.0	62.3	58.7
Prepelvic distance (PPLD)	35.0	40.2	35.7
Prepectoral distance (PPCD)	21.5	24.5	21.8
Caudal peduncle depth (CPD)	5.1	6.8	6.6
Caudal peduncle length (CPL)	16.4	18.0	18.6
Head width (HW)	10.1	10.7	11.0
Pectoral-fin length (PCFL)	18.8	18.5	17.8
Pelvic-fin length (PLFL)	11.6	10.9	12.7
Pelvic-anal-fin distance (DPLAF)	19.8	22.9	22.3
Pectoral-anal-fin distance (DPCAF)	33.9	37.8	37.3
Anal-fin base length (AFL)	29.5	22.1	24.3
Dorsal-fin base length (DFL)	23.2	19.4	20.0
**Percent of HL**			
Snout length (SNL)	26.6	26.3	23.1
Post-orbital length (POL)	59.0	59.6	59.5
Head width (HW)	46.1	47.5	47.7
Interorbital width (IOW)	33.2	31.9	34.5
Head depth (HD)	79.4	78.9	78.7
Eye diameter (ED)	19.5	19.9	19.0
Inter-nostril distance (DNN)	4.7	4.2	5.4
Nares-eye distance (DNE)	7.2	5.9	3.1
Mouth width (MW)	14.8	15.8	11.8
Ratios and angles			
Inter-orbital width as % head width	72.0	67.2	72.3
Pre-anal distance/pre-dorsal distance	87.8	100.0	93.0
Inter-nostril width as % interorbital width	14.2	13.1	15.6
Caudal peduncle depth as % CP length	31.2	37.7	35.3
Length of dorsal/length of anal	78.5	87.7	82.5
**Counts**			
Dorsal rays (simple+branched)	3+21=24	3+18=21	3+17=20
Anal rays (simple +branched)	3+28=31	3+21=24	3+22=25
Anal rays before dorsal	7	2	4
Anal rays beyond last dorsal ray	3	2	2
Pectoral rays	10	10	10
Pelvic rays	6	6	6
Total midlateral scales (pierced+unpierced)	43+2=45	42+2=44	42+2=44
Rows scales above lateral line to dorsal	9	9	7
Rows scales below lateral line to pelvic	9	9	10
Caudal peduncular scales	12	12	12
Teeth upper jaw/lower jaw	5/6	5/6	5/6
Total vertebrae	43	41	40
Hypurals 1 & 2	unfused	unfused	unfused

#### Color.

In life, a light cinnamon brown along sides, darker along dorsum, reflective coppery pigment on lower half of head and along belly to anal fin, mouth and chin whitish. Eye with golden iris, dark center. Faint, diffuse broad band of pigment between dorsal and anal fin occupying 4–5 scales, darker above. Numerous depigmented spots over electroreceptors conspicuous on snout, top of head, belly and upper back. Fins with very lightly pigmented rays, interradial membranes hyaline. In preservation, light brown.

#### Electric organ discharge.

EOD waveform recorded from the holotype specimen is very short, about 0.55 millisecond total duration (recorded at 23.2 °C) if measured from the onset of the first very weak head-negative phase (P0) and only 0.28 milliseconds if measured from the rising phase of the head-positive phase (P1) to the end of the large head negative phase (P2) using a 2% departure-from-baseline threshold (Fig. 4A). P1 is twice the duration and 85% of the amplitude of P2. Both P1 and P2 rise and fall smoothly with no inflection points. In advance of P1 there is a weak head-negative (P0) phase, only 0.2% of the peak-to-peak height of P1 and P2, difficult to see without additional amplification. The presence of a P0 indicates that the electrocytes of this species’ electric organ have penetrating stalks innervated on the posterior side of the electrocyte (type Pa) unlike the non-penetrating stalk electrocytes (type NPp) of species of sister genera *Boulengeromyrus* and *Ivindomyrus* (Sullivan et al. 2000). The power spectrum of the EOD (Fig. 4B) exhibits a broad plateau between 1700 and 8200 Hz with peak power at 4300 Hz.

#### Etymology.

The specific epithet is a Latinized noun in the genitive case and refers to the Ogooué River of Gabon.

#### Distribution and ecology.

Currently known only from the Ogooué River at Doumé falls. At the collection site, we recorded a water temperature of 26.7 °C, a pH of 6.89, water conductivity of 13.8 μS/cm and dissolved oxygen of 84.7%.

### Cryptomyrus ona sp. n.

Taxon classificationAnimaliaOsteoglossiformesMormyridae

http://zoobank.org/D75443F1-97B6-4A89-B1C4-EC06167D47A5

**Figure 8.**
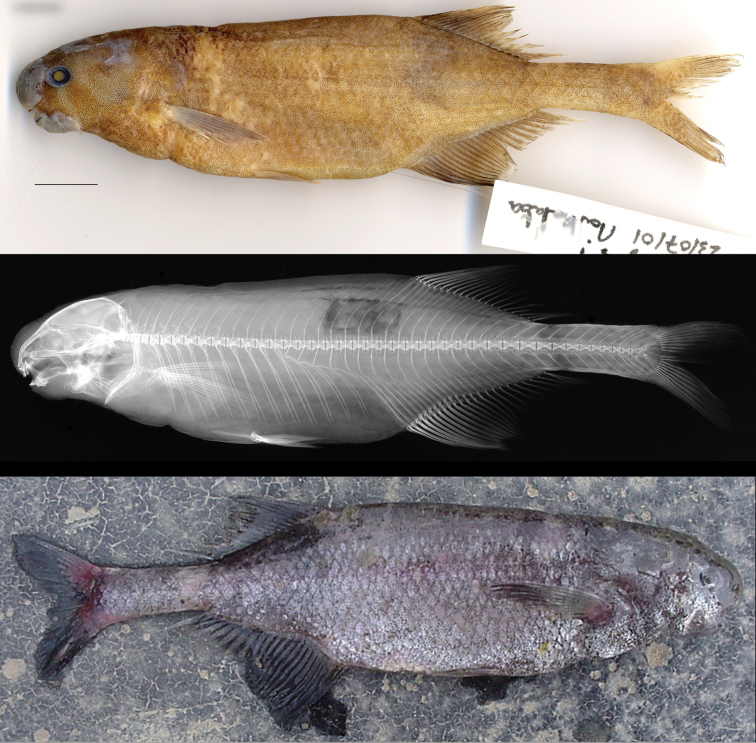
Holotype of *Cryptomyrus ona* MNHN 2003-0425, male, 110 mm SL, Moukalaba River near confluence with Nyanga River, Nyanga Province, Gabon. Preserved specimen shown above radiograph and photo of specimen shortly after collection. Scale bar = 1 centimeter.

**Figure 9.**
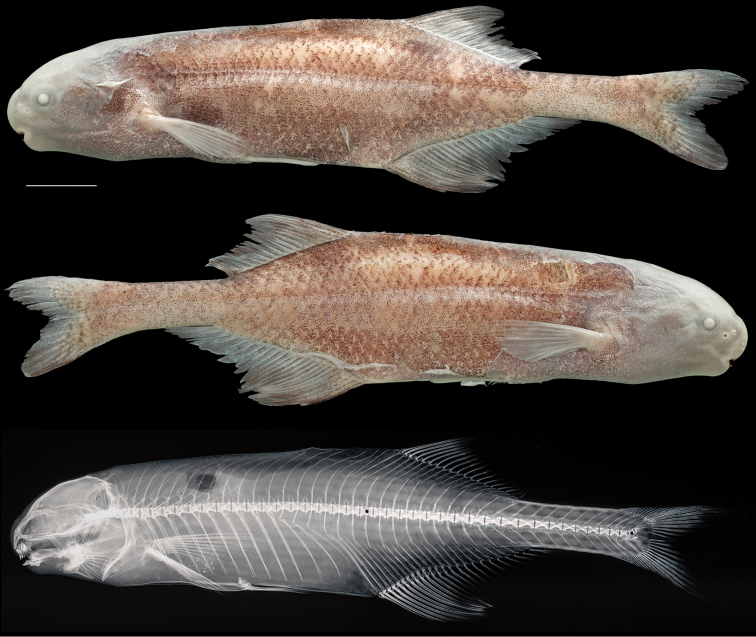
Non-type specimen of *Cryptomyrus ona* CUMV 98647, male, 98 mm SL, Mabounié River, tributary of Ngounié River, Ogooué River basin, Ngounié Province, Gabon. Left and right views of preserved specimen above radiograph. Scale bar = 1 centimeter.

Figs 8 , 9 ; Table 4

#### Holotype.

MNHN 2003-0425, 120.6 mm TL, 107.8 SL, male; Nyanga Province, Gabon: Moukalaba River very near its confluence with the Nyanga River, just above ferry landing on Tchibanga-Digoudou road, 2°47.3400'S, 10°43.7160'E, gill net at night, S. Lavoué & V. Mamonekene, 23 July 2001.

#### Other (non-type) specimen.

CUMV 98647, 115.1 mm TL, 98.1 mm SL, male; Ngounié Province, Gabon: Mabounié River (Lower Ngounié-Ogooué River basin), Station HBG-010, 0°45.1692'S, 010°32.9202'E, gill net at night, Y. Fermon, J.H. Mve Beh, & J.D. Mbega, 21 February 2012.

#### Diagnosis.

*Cryptomyrus ona* sp. n. is readily differentiated from its sole congener, *Cryptomyrus ogoouensis* sp. n., in having an anal-fin origin located only just in advance of the dorsal fin (first dorsal ray above anal-fin ray 3 vs. first dorsal-fin ray above anal-fin ray 7), a deep caudal peduncle (6.0–6.8% SL vs. 5.1% SL), and lobes of caudal fin markedly shorter than caudal peduncle (vs. nearly as long as peduncle).

#### Description.

Morphometric and meristic data for holotype (male, 107.8 mm SL) presented in Table 4. Maximum size of this species unknown. Body fusiform with dorsal and ventral profiles gently convex, greatest body depth between terminus of pelvic fin and urogenital pore. Body compressed, widest at head. Dorsal head profile gently convex with shallow slope downwards to snout, slight inflection between snout and head above eye. Ventral profile of head with marked concavity between gular region and chin, with body depth increasing rapidly from here to below pelvic fin origin. Snout bulbous, rounded, tip below horizontal through center of eye, projecting above upper lip. Nostrils well separated from each other and from eye; posterior naris at level of bottom of orbit, anterior naris below line through center of eye; straight line drawn through nostrils passes through tip of snout and just below pectoral-fin origin. Mouth subinferior, small; rictus below posterior naris, in advance of eye. Chin swelling modest, but broad, nonprotrusive. Premaxillary teeth 5, strongly notched, dentary teeth 6, spatulate, center four oriented horizontally, center two with broad contact between inner edges which are longer than outer edges, projecting well beyond neighboring pair to which they are closely appressed, trowel-like (Fig. 6). Eye large, laterally positioned on head. Predorsal distance equal to preanal distance; anal fin only slightly in advance of dorsal fin: first dorsal-fin ray above first or second branched anal-fin ray; dorsal and anal fins with short bases. Dorsal-fin rays 3+18, anal-fin rays 3+21; 2 anal-fin rays beyond last dorsal-fin ray. Dorsal fin with falcate posterior margin. Longest dorsal-fin ray is first branched ray; next 10 rays successively shorter; next 5 rays approximately equal in length, final 2 rays longer. Anal-fin ray with gently falcate posterior margin. Longest anal-fin ray is second branched ray; next 10 rays successively shorter, next 6 rays more or less equal in length, final 3 successively longer. Pectoral fin with 10 rays, tip pointed, extending beyond the origin of the pelvic fin, but short of halfway. Pelvic fin with 6 rays, closer to pectoral than to anal. Caudal peduncle deep, depth at middle of peduncle slightly deeper than at origin. Caudal fin deeply forked with 20 rays in each lobe, lobes short, scaled at their bases with bluntly pointed ends; distance from caudal flexion to caudal tips shorter than CPL. Body scales thin, cycloid, head naked. Body scales large along sides, smaller dorsally: 42 pierced lateral line scales + 2 unpierced along midlateral line, 18 scales in transverse series between origins of dorsal and anal fins, 9 from pelvic fin to midlateral line, 12 around caudal peduncle. Total vertebrae 41, epineurals associated with vertebrae 1–8, pleural ribs directly attached to vertebral centra 2–13, ribs displaced beneath haemal arches on vertebrae 14–17, caudal vertebrae 18–41. Hypurals 1 & 2 unfused.

#### Color.

Photo of holotype specimen recovered from gill net, several hours post-mortem, shows a purplish-gray body, darker along dorsum, with whitish marbling/speckling on lower half of head and along belly to anal fin and diffuse band of pigment below dorsal fin occupying 4 scales, darker above, lighter or absent above anal fin. Numerous depigmented spots over electroreceptors conspicuous on snout, top of head, belly and upper back. Fins with darkly pigmented rays, interradial membranes hyaline. In preservation, body yellowish tan.

#### Electric organ discharge.

Unknown.

#### Etymology.

The specific epithet is a noun in apposition that honors Marc Ona Essangui, Gabonese environmental and civic activist, founder and executive director of the NGO Brainforest and recipient of the 2009 Goldman Environmental Prize, in appreciation for his efforts to protect Gabon’s equatorial forests and wetlands.

#### Distribution and ecology.

Known from the type locality in the Moukalaba River at its confluence with the Nyanga River. A second specimen referred to this species comes from the Mabounié River, a small right-bank affluent of the lower Ngounié River, below Samba Falls. Both specimens were caught by gill net at night. At the type locality we recorded water temperature of 23.6 °C, pH of 8.0, water conductivity of 116.0 μS/cm and dissolved oxygen of 4.98 mg/l. Water conductivity at the Mabounié River at time of collection of that specimen was 48 μS/cm.

### Revised key to the mormyrid genera of Lower Guinea, West-Central Africa.

Lower Guinea is defined as the Atlantic drainages of Africa from the Cross River of Cameroon in the north to the Chiloango River of Cabinda/D.R. Congo in the south (Roberts 1975). This artificial key is modified after Hopkins et al. (2007) in which some character states are illustrated. Distribution of each genus within Lower Guinea is indicated in parentheses.

**Table:** 

1	Nostrils close to one another and to the eye; mouth inferior, below the level of the eye; body short and rather deep	**genus *Petrocephalus*** (subfamily Petrocephalinae; widespread)
–	Nostrils separated from each other and from the eye; mouth terminal or inferior, in advance of the eye, body deep or elongate	(subfamily Mormyrinae)
2	Teeth extending along the entire edge of both jaws in a single series, 10–36 in each jaw; mouth terminal, well in advance the eye; body elongate, depth more than 5.2 times into SL	***Mormyrops*** (widespread)
–	Teeth restricted to middle of each jaw, 3–10 in each jaw	**3**
3	Dorsal fin more than twice the length of anal, originating in advance of pelvic fin insertion	***Mormyrus*** (Cross, Sanaga, Nyong)
–	Dorsal fin 0.35–1.25 times the length of the anal, originating behind pelvic fin insertion	**4**
4	Pelvic fin insertion closer to the anal than to the pectoral fins; body very elongate, at least 8–11 times as long as deep	***Isichthys*** (widespread)
–	Pelvic fin insertion mid-way between anal and pectoral fins or closer to pectorals; body less elongate to short	**5**
5	Posterior nostril located close to the border of the mouth	***Stomatorhinus*** (Ogooué, Ivindo, Kouilou-Niari)
–	Neither nostril close to the border of the mouth	**6**
6	Snout elongated and tubular, its length greater than the post-orbital length of the head; snout turned downward	***Campylomormyrus*** (Sanaga)
–	Snout non-tubular, its length less than the post-orbital length of the head	**7**
7	Prominent tapered cylindrical barbel-like appendage under the chin, extending forward from below lower jaw	***Gnathonemus*** (Cross, Sanaga)
–	Submental appendage reduced to fleshy swelling or absent altogether	**8**
8	Submental appendage prominent, extending slightly beyond the end of the upper jaw, mouth terminal	***Marcusenius*** (widespread)
–	Submental appendage not extending beyond end of upper jaw or absent altogether, mouth terminal or inferior	**9**
9	Scales along midlateral line 44 or 45, broad submental swelling, mouth subinferior	***Cryptomyrus*** (Ogooué, Nyanga)
–	Scales along midlateral line 47 or more, chin swelling only slightly developed or absent; mouth terminal or inferior	**10**
10	Dorsal and anal fins approximately equal in length and originating at the same vertical level, dorsal fin with 31–34 rays, anal fin with 31–35 rays, mouth inferior	***Hippopotamyrus*** (Cross, Sanaga, Wouri, Lokoundjé)
–	Dorsal fin shorter than anal fin and with fewer than 30 rays	**11**
11	Body moderately elongate, depth 18–22% SL	**12**
–	Body moderately deep, more than 23% SL	**13**
12	Anal and dorsal fins terminate at about the same level. Distal tips of last anal and dorsal rays not offset	***Paramormyrops*** (widespread)
–	Anal fin terminates beyond the end of dorsal. Distal tips of last anal and dorsal fin rays offset	***Brienomyrus*** (widespread)
13	Mouth terminal	**14**
–	Mouth subterminal	**15**
14	Snout straight, short and blunt, no darkly pigmented transverse band between dorsal and anal fins	***Brevimyrus*** (Cross)
–	Snout turned downward, long, conical; darkly pigmented transverse band between dorsal and anal fins	***Boulengeromyrus*** (Ntem, Ivindo)
15	Posterior nostril closer to anterior nostril than to eye; darkly pigmented transverse band between dorsal and anal fins	***Ivindomyrus*** (Ntem, Ivindo, Ogooué, Nyanga)
–	Posterior nostril closer to eye than to anterior nostril; no darkly pigmented transverse band between dorsal and anal fins	***Pollimyrus*** (Cross, Wouri, Kouilou-Niari)

## Discussion

*Cryptomyrus* is the first new genus of Mormyridae to be described since *Paramormyrops*, from the same region of Africa, in the late 1970s (Taverne et al. 1977). Given the perception that Gabon is better sampled for fishes than other parts of Central Africa (Stiassny and Hopkins 2007; Stiassny et al. 2011), having no more than three individuals of this mormyrid lineage in collections may seem surprising. In reality, only a small percentage of Gabon’s aquatic environments have been visited by ichthyologists. Tropical freshwaters harbor a high proportion of narrowly distributed fish species and in poorly inventoried regions like Gabon such species may long go undetected (Pelayo-Villamil et al. 2014). We have too few data to infer whether these two *Cryptomyrus* species are rare throughout their range, or simply rare where ichthyologists have chosen to collect (Hercos et al. 2012). Because of the “commonness of rarity” in the tropics, description of singletons remains a common taxonomic practice (Lim et al. 2012). Any rule requiring a minimum number of specimens to erect a new taxon would leave a significant proportion of tropical biodiversity undocumented, including taxa most at risk of extinction.

The Moukalaba-Nyanga system of Gabon, the type locality of *Cryptomyrus ona*, remains understudied and is likely to produce additional taxonomic novelties for Mormyridae and other groups. Doumé, the Ogooué River collection site for *Cryptomyrus ogoouensis*, is already an important type locality for fishes. From collections made here in 1876–77 by Alfred Marche, naturalist on the first of Pierre Savorgnan de Brazza’s expeditions that explored the sources of the Ogooué (see Sullivan 2007), Henri Émile Sauvage described the mormyrids *Mormyrops sphekodes* (now *Paramormyrops sphekodes*), *Petrocephalus marchei* (now *Ivindomyrus marchei*) and *Petrocephalus simus* along with five non-mormyrid species that remain valid today (Sauvage 1879, 1880). Until our two brief collecting trips in 2011 and 2014, Doumé had apparently not been revisited by ichthyologists. Our intention was to clarify the identity of *Paramormyrops sphekodes*, a taxon whose name is often erroneously applied. In the course of making new collections of *Paramormyrops sphekodes* from Doumé in 2014, we collected the single *Cryptomyrus ogoouensis* specimen as well as specimens of a new *Paramormyrops* being described separately.

Despite its provenance from a part of the greater Ogooué basin, not the Nyanga basin, we treat the Mabounié River specimen as a non-type specimen of *Cryptomyrus ona* on the basis of its morphological and genetic similarity to the *Cryptomyrus ona* type specimen. It is worth noting that headwaters of the Ngounié abut those of the Nyanga and that at least two other fish species, *Synodontis ngouniensis* De Weirdt, Vreven & Fermonm, 2008 and *Aphyosemion primigenium* Radda & Huber, 1977 appear to have exclusive distribution in these two river basins, having never been collected elsewhere in the wider Ogooué system. *Synodontis punu* Vreven & Milondo, 2009 is found in these two river basins plus the Kouilou-Niari basin to the south within the Republic of Congo.

The distinctive morphology of these specimens drew our attention to them, but provided few clues about their affinities to other mormyrid species. While we have an EOD waveform recording only from the Doumé specimen, its uniqueness among those known from Gabon’s mormyrids also helped to highlight its special status. Sequence data were necessary for confirming that these three individuals are indeed closest relatives and for placing them phylogenetically within the Mormyrinae. The combination of these datasets– morphology, electric signals and DNA– provides a practical, integrative, and evolutionary framework in which to evaluate the status of candidate mormyrid species. The utility of this approach has been demonstrated in a number of other recent publications on Mormyridae (e.g. Kramer and Swartz 2010; Lavoué et al. 2010; Lavoué and Sullivan 2014).

Morphological synapomorphies remain to be discovered for the “Lower Guinea Clade” of Mormyridae to which *Cryptomyrus* belongs (Fig. 5), a group that has been recognized from a series of molecular phylogenetic studies (Alves-Gomes and Hopkins 1997; Lavoué et al. 2000, 2003; Sullivan et al. 2000, 2002, 2004). All are species endemic to the river systems of Lower Guinea with the exception of two species of *Paramormyrops* described from the Congo basin and a third species (*Paramormyrops kingsleyae*) common to the Ogooué and the Congo (Sullivan et al. 2002; Hopkins et al. 2007). A fourth species of *Paramormyrops*, *Paramormyrops jacksoni* (Poll, 1967) from the upper Zambesi basin of Angola, has never been sequenced and is likely misclassified. In the Lower Guinea watersheds, mormyrid species of Lower Guinea Clade genera *Paramormyrops*, *Ivindomyrus*, *Boulengeromyrus* and *Cryptomyrus* appear in some ways to be eco-morphological equivalents to species of *Mormyrus*, *Marcusenius*, *Cyphomyrus*, and *Pollimyrus* that occur the Congo and Nilo-Sudanic drainages. *Cryptomyrus ogoouensis* and *Cryptomyrus ona* represent only a small portion of the unrecognized diversity within the Lower Guinea Clade of Mormyridae as more than a dozen species of *Paramormyrops* from Gabon still await description.

Biodiversity unknown to science is invisible to conservation efforts. The rivers of Gabon, like others across Africa, are increasingly impacted by logging, road-building, mining, and hydropower dam construction (Brummett et al. 2011). Grand Poubara Dam on the upper Ogooué River is already completed and construction may begin soon on dams on the Okano and Ngounié Rivers (Ministry of Energy and Water Resources of Gabon 2015). The discovery of *Cryptomyrus ogoouensis* and *Cryptomyrus ona* from just three specimens collected over 13 years reveals the incompleteness of our knowledge of this fish fauna and should motivate us to finish the task of documenting it before human activity further alters these ecosystems.

### Comparative material examined

*Cyphomyrus psittacus*: D.R. Congo, Orientale, Wagenia Falls, CUMV 97543, 4, 88–133 mm SL, tag nos. JPS-0537, JPS-0538, 2 untagged.

*Hippopotamyrus castor*: Cameroon, Centre, Sanaga R. at Nachtigal Falls, CUMV 89955, 6, 104–136 mm SL, tag nos. 6018, 6019, 6020, 6037, 6038, 6039.

*Ivindomyrus marchei*: Gabon, Moyen-Ogooué, Ogooué R. at Lambaréné, CUMV 80252, 2, 113 & 115 mm SL, tag nos. 2940, 2941; CUMV 80244, 1, 115 mm SL, tag no. 2908; Ngounié, Loétsi R., CUMV 95128, 3, 105–114 mm SL, tag nos. 6665, 6673, 6674; Ogooué-Ivindo, Ivindo R. at Loa Loa, CUMV 96827, 4, 90–133 mm SL, tag nos. JPS-1101, JPS-1101, JPS-1038, JPS-1043, JPS-1086; Ogooué-Lolo, Sébé R., CUMV uncat., 1, 105 mm SL, field no. TNC14-061; CUMV 89325, 1, 95 mm SL, tag no. 5668.

*Ivindomyrus opdenboschi*: Gabon, Ogooué-Ivindo, Ivindo R. at Loa Loa, CUMV 83107, 2, 89 & 97 mm SL, tag nos. 4800, 4847; CUMV 89324, 4, 101–114 mm SL, tag no. 5599, 5653, 5654, 5682.

*Marcusenius moorii*: Gabon, Haut-Ogooué, Ogooué R., CUMV 80460, 1, 123 mm SL, tag no. 3453; Okoloville, CUMV 81623, 1, 124 mm SL, no tag; Moyen-Ogooué, Ogooué R. off point of Lambaréné Island, CUMV 83100, 4, 111–125 mm SL, tag nos. 4950, 4951, 4954, 4972; Ngounié, Louétsi R., CUMV 80346, 1, 126 mm SL, tag no. 3107; CUMV 84517, 1, 122 mm SL, tag no. 2693; Ogooué-Ivindo, Ivindo R., CUMV 89372, 2, 100 & 115 mm SL, tag nos. 5556, 5576; CUMV 96830, 1, 107 mm SL, tag no. JPS-1020; CUMV 81658, 1, 112 mm SL, tag no. 2082; Ogooué R. at Lopé, CUMV 83084, 2, 85 & 85 mm SL, tag nos. 4760, 4761.

*Paramormyrops kingsleyae*: Gabon, Moyen-Ogooué, Lambaréné, Mikouma Creek, CUMV 80232, 8, 86–108 mm SL, tag nos. 2846–2851, 2854, A3; Ngounié, Biroundou Creek, CUMV 80342, 2, 94 & 121 mm SL, tag nos. 3214, 3215; Bambomo Creek, CUMV 80343, 2, 102 & 109 mm SL, tag nos. 3121, 3128; Woleu-Ntem, Bikagala Creek, CUMV 80348, 2, 102 & 118 mm SL, tag nos. 3209, 3210; CUMV 80251, 7, 85–118 mm SL, tag nos. 3123, 3125, 3129, 3192, 3196, 3197, 3199; Bakanda Creek CUMV 80527, 3, 93–120 mm SL, tag nos. 3221, 3223, 3224.

*Paramormyrops* sp. (undescribed species): Gabon, Haut-Ogooué, Okoloville, CUMV 80474, 3, 84–101 mm SL; CUMV 80812, 8, 73–99 mm SL; Creek at Bibassa, MRAC 9450 P 0074, 1, 95 mm SL; Ogooué-Ivindo, Balé Creek, CUMV 83110, 1, 90 mm SL; Ivindo R. at Loa Loa rapids, CU89380, 3, 87–115 mm SL.

*Paramormyrops* sp. “SN4”: Gabon, Haut-Ogooué, Ogooué R. at Franceville, CUMV 80458, 1, 127 mm SL, tag no. 3396; CUMV 80463, 2, 126 & 126 mm SL, tag nos. 3466, 3477; CUMV 80901, 2, 118 & 125 mm SL, tag nos. AG, AH; Mpassa R., CUMV 80501, 3, 112–121 mm SL, tag nos. 3747, 3750, 3755; CUMV 80507, 2, 127, 128 mm SL, tag nos. 3707, 3710; Ogooué-Lolo, Doumé, CUMV 96811, 2, 108 & 117 mm SL, tag nos. JPS-1117, JPS-1119; Woleu-Ntem, Okano R. at Na, CUMV 83059, 3, 86–106 mm SL, tag nos. 4680, 4681, 4684; CUMV 83078, 2, 85 & 86 mm SL, tag nos. 4728, 4729; CUMV 83085, 2, 85 & 87 mm SL, tag nos. 4723, 4725; CUMV 83155, 1, 103 mm SL, tag no. 5.

*Stomatorhinus walkeri*: Gabon, Ogooué-Lolo, Ogooué R., Haut-Ogooué, Ogooué R., CUMV 80477, 2, 86 & 89 mm SL, tag nos. 3605. 3608; CUMV 81631, 1, 79 mm SL, no tag. Ogooué-Lolo, Doumé, MNHN A-0894 (holotype of *Petrocephalus affinis* Sauvage), 1, 82 mm SL. Moyen-Ogooué, Ogooué R., BMNH 1867.5.3.15, .16 (syntypes of *Mormyrus walkeri* Günther), 2, 85, 86 mm SL; CUMV 80237, 2, tag nos. 2877, 2880; CUMV 80248, 1, 82 m mSL, tag no. 2912; CUMV 80255, 1, 75 mm SL, tag no. 2922; Ogooué-Ivindo, Ogooué R. at Lopé, CUMV 83071, 1, 80 mm SL, tag no. 4770.

## Supplementary Material

XML Treatment for Cryptomyrus

XML Treatment for Cryptomyrus ogoouensis

XML Treatment for Cryptomyrus ona
